# Automatic Brain Tumor Segmentation Based on Cascaded Convolutional Neural Networks With Uncertainty Estimation

**DOI:** 10.3389/fncom.2019.00056

**Published:** 2019-08-13

**Authors:** Guotai Wang, Wenqi Li, Sébastien Ourselin, Tom Vercauteren

**Affiliations:** ^1^School of Mechanical and Electrical Engineering, University of Electronic Science and Technology of China, Chengdu, China; ^2^School of Biomedical Engineering and Imaging Sciences, King's College London, London, United Kingdom; ^3^NVIDIA, Cambridge, United Kingdom

**Keywords:** brain tumor segmentation, deep learning, uncertainty, data augmentation, convolutional neural network

## Abstract

Automatic segmentation of brain tumors from medical images is important for clinical assessment and treatment planning of brain tumors. Recent years have seen an increasing use of convolutional neural networks (CNNs) for this task, but most of them use either 2D networks with relatively low memory requirement while ignoring 3D context, or 3D networks exploiting 3D features while with large memory consumption. In addition, existing methods rarely provide uncertainty information associated with the segmentation result. We propose a cascade of CNNs to segment brain tumors with hierarchical subregions from multi-modal Magnetic Resonance images (MRI), and introduce a 2.5D network that is a trade-off between memory consumption, model complexity and receptive field. In addition, we employ test-time augmentation to achieve improved segmentation accuracy, which also provides voxel-wise and structure-wise uncertainty information of the segmentation result. Experiments with BraTS 2017 dataset showed that our cascaded framework with 2.5D CNNs was one of the top performing methods (second-rank) for the BraTS challenge. We also validated our method with BraTS 2018 dataset and found that test-time augmentation improves brain tumor segmentation accuracy and that the resulting uncertainty information can indicate potential mis-segmentations and help to improve segmentation accuracy.

## 1. Introduction

In adults, gliomas are the most common primary brain tumors. They begin in the brain's glial cells and are typically categorized into different grades: High-Grade Gliomas (HGG) grow rapidly and are more malignant, while Low-Grade Gliomas (LGG) are slower growing tumors with a better patient prognosis (Louis et al., 2016). Magnetic Resonance Imaging (MRI) of brain tumors is critical for progression evaluation, treatment planning and assessment of this disease. Different sequences of MRI can be used for brain tumor imaging, such as T1-weighted, T2-weighted, contrast enhanced T1-weighted (T1ce), and Fluid Attenuation Inversion Recovery (FLAIR) images. T2 and FLAIR images mostly highlight the whole tumor region (including infiltrative edema), and T1 and T1ce images give a better contrast for the tumor core region (not including infiltrative edema) (Menze et al., 2015). Therefore, these different sequences providing complementary information can be combined for the analysis of different subregions of brain tumors.

Segmenting brain tumors and subregions automatically from multi-modal MRI is important for reproducible and accurate measurement of the tumors, and this can assist better diagnosis, treatment planning and evaluation (Menze et al., 2015; Bakas et al., 2017b). However, it remains difficult for automatic methods to accurately segment brain tumors from multi-modal MRI. This is due to the fact that the images often have ambiguous boundaries between normal tissues and brain tumors. In addition, though prior information of shape and position has been used for segmentation of anatomical structures such as the liver (Wang et al., 2015) and the heart (Grosgeorge et al., 2013), the shape, size and position of brain tumors have considerable variations across different patients. This makes it difficult to use a prior shape and position for robust segmentation of brain tumors. Recently, deep learning methods with Convolutional Neural Networks (CNNs) have become the state-of-the-art approaches for brain tumor segmentation (Bakas et al., 2018). Compared with traditional supervised learning methods such as decision trees (Zikic et al., 2012) and support vector machines (Lee et al., 2005), CNNs can learn the most useful features automatically, without the need for manual design and selection of features.

A key problem for CNN-based segmentation is to design a suitable network structure and training strategy. Using a 2D CNN in a slice-by-slice manner has a relatively low memory requirement (Havaei et al., 2016), but the network ignores 3D information, which will ultimately limit the performance of the segmentation. Using 3D CNNs can better exploit 3D features, but requires a large amount of memory, which may limit the input patch size, depth or feature numbers of the CNNs (Kamnitsas et al., 2017b). As a trade-off, 2.5D CNNs can take advantage of inter-slice features compared with 2D CNNs and have a lower memory requirement than their 3D counterparts. In addition, whole tumor, tumor core and enhancing tumor core follow a hierarchical structure. Using the segmentation of whole tumor (tumor core) to guide the segmentation of tumor core (enhancing tumor core) can help to reduce false positives. Therefore, in this work, we propose a framework consisting of a cascade of 2.5D networks for brain tumor segmentation from multi-modal 3D MRI that achieves a trade-off between memory consumption, model complexity and receptive field.

For medical images, uncertainty information of segmentation results is important for clinical decisions as it can help to understand the reliability of the segmentations (Shi et al., 2011) and identify challenging cases necessitating expert review (Jungo et al., 2018). For example, for brain tumor images, the low contrast between surrounding tissues and the segmentation target leads voxels around the boundary to be labeled with less confidence. The uncertainty information of these voxels can indicate regions that have potentially been mis-segmented, and therefore can be employed to guide interactions of human to refine the segmentation results (Wang et al., 2018b). In addition, compared with datasets for natural image recognition (Russakovsky et al., 2015), datasets for CNN-based medical image segmentation methods are relatively small, which tends to result in more uncertain predictions in the segmentation outputs, and can lead to structure-wise uncertainty for downstream tasks, such as measuring the volume of tumor regions. Therefore, this work also aims at providing voxel-wise and structure-wise uncertainty information for CNN-based brain tumor segmentation. Unlike model-based (*epistemic*) uncertainty obtained by test-time dropout (Gal and Ghahramani, 2016; Jungo et al., 2017, 2018), we investigate image-based (*aleatoric*) uncertainty obtained by test-time augmentation that has previously been mainly used for improving segmentation accuracy (Matsunaga et al., 2017; Radosavovic et al., 2018).

This paper is a combination and an extension of our previous works on brain tumor segmentation (Wang et al., 2017, 2018a), where we proposed a cascade of CNNs for sequential segmentation of brain tumor and the subregions from multi-modal MRI, which decomposes the complex task of multi-class segmentation into three simpler binary segmentation tasks. We also proposed 2.5D network structures with anisotropic convolution for the segmentation task as a result of trade-off between memory consumption, model complexity and receptive field. In this paper, we extend them in two aspects. First, we use test-time augmentation to obtain uncertainty estimation of the segmentation results, and additionally propose an uncertainty-aware conditional random field (CRF) for post-processing. The results show that uncertainty estimation not only helps to identify potential mis-segmentations but also can be used to improve segmentation performance. Both voxel-level and structure-level uncertainty are analyzed in this paper. Second, we implement more ablation studies to demonstrate the effectiveness of our segmentation pipeline.

## 2. Related Works

### 2.1. Brain Tumor Segmentation From MRI

Existing brain tumor segmentation methods include generative and discriminative approaches. By incorporating domain-specific prior knowledge, generative approaches usually have good generalization to unseen images, as they directly model probabilistic distributions of anatomical structures and textural appearances of healthy tissues and the tumor (Menze et al., 2010). However, it is challenging to precisely model probabilistic distributions of brain tumors. In contrast, discriminative approaches extract features from images and associate the features with the tissue classes using discriminative classifiers. They often require a supervised learning set-up where images and voxel-wise class labels are needed for training. Classical methods of this category include decision trees (Zikic et al., 2012) and support vector machines (Lee et al., 2005).

Recently, CNNs as a type of discriminative approach have achieved promising results on multi-modal brain tumor segmentation tasks. Havaei et al. (2016) combined local and global 2D features extracted by a CNN for brain tumor segmentation. Although it outperformed the conventional discriminative methods, the 2D CNN only uses 2D features without considering the volumetric context. To incorporate 3D features, applying the 2D networks in axial, sagittal and coronal views and fusing their results has been proposed (McKinley et al., 2016; Li and Shen, 2017; Hu et al., 2018). However, the features employed by such a method are from cross-planes rather than entire 3D space.

DeepMedic (Kamnitsas et al., 2017b) used a 3D CNN to exploit multi-scale volumetric features and further encoded spatial information with a fully connected Conditional Random Field (CRF). It achieved better segmentation performance than using 2D CNNs but has a relatively low inference efficiency due to the multi-scale image patch-based analysis. Isensee et al. (2018) applied 3D U-Net to brain tumor segmentation with a carefully designed training process. Myronenko (2018) used an encoder-decoder architecture for 3D brain tumor segmentation and the network contained an additional branch of variational auto-encoder to reconstruct the input image for regularization. To obtain robust brain tumor segmentation resutls, Kamnitsas et al. (2017a) proposed an ensemble of multiple CNNs including 3D Fully Convolutional Networks (FCN) (Long et al., 2015), DeepMedic (Kamnitsas et al., 2017b), and 3D U-Net (Ronneberger et al., 2015; Abdulkadir et al., 2016). The ensemble model is relatively robust to the choice of hyper-parameters of each individual CNN and reduces the risk of overfitting. However, it is computationally intensive to run a set of models for both training and inference (Malmi et al., 2015; Pereira et al., 2017; Xu et al., 2018).

### 2.2. Uncertainty Estimation for CNNs

Uncertainty information can come from either the CNN models or the input images. For model-based (*epistemic*) uncertainty, exact Bayesian modeling is mathematically grounded but often computationally expensive and hard to implement. Alternatively, Gal and Ghahramani (2016) cast test-time dropout as a Bayesian approximation to estimate a CNN's model uncertainty. Zhu and Zabaras (2018) estimated uncertainty of a CNN's parameters using approximated Bayesian inference via stochastic variational gradient descent. Other approximation methods include Monte Carlo batch normalization (Teye et al., 2018), Markov chain Monte Carlo (Neal, 2012) and variational Bayesian (Louizos and Welling, 2016). Lakshminarayanan et al. (2017) proposed a simple and scalable method using ensembles of models for uncertainty estimation. For test image-based (*aleatoric*) uncertainty, Ayhan and Berens (2018) found that test-time augmentation was an effective and efficient method for exploring the locality of a test sample in *aleatoric* uncertainty estimation, but its application to medical image segmentation has not been investigated. Kendall and Gal (2017) proposed a unified Bayesian framework that combines *aleatoric* and *epistemic* uncertainty estimations for deep learning models. In the context of brain tumor segmentation, Eaton-Rosen et al. (2018) and Jungo et al. (2018) used test-time dropout to estimate the uncertainty. Wang et al. (2019a) analyzed a combination of *epistemic* and *aleatoric* uncertainties for whole tumor segmentation, but the uncertainty information of other structures (tumor core and enhancing tumor core) was not investigated.

## 3. Methods

### 3.1. Segmentation Pipeline and Network Structure

#### 3.1.1. Triple Cascaded Framework

**Figure 1:**
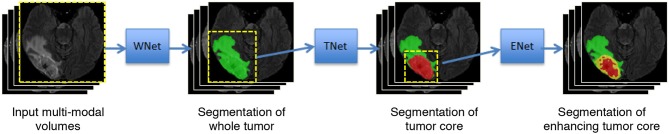
Our proposed framework with triple cascaded CNNs for multi-modal brain tumor segmentation. We use three CNNs to hierarchically and sequentially segment whole tumor, tumor core and enhancing tumor core, and the CNNs are referred to as WNet, TNet, and ENet, respectively.

Malmi et al. (2015) and Pereira et al. (2017) used a cascade of two stages to segment brain tumors where the whole tumor was segmented in the first stage and then all substructures were segmented in the second stage. To better take advantage of the hierarchical property of brain tumor structures, in our preliminary study (Wang et al., 2017), we proposed a cascade of three CNNs to hierarchically and sequentially segment the whole brain tumor, tumor core and enhancing tumor core, which is followed by some more recent works (Ma and Yang, 2018; Xu et al., 2018). As shown in Figure 1, we use three networks (WNet, TNet, and ENet) to segment these structures, respectively. First, the whole tumor is segmented by WNet. Then the input multi-modal image is cropped according to the bounding box of the segmented whole tumor. Second, TNet segments the tumor core from the cropped image region, and the input image is further cropped based on the bounding box of the segmented tumor core. Finally, the enhancing tumor core is segmented by ENet from the second cropped region. We use the segmentation result of whole tumor (tumor core) as a crisp mask for the result of tumor core (enhancing tumor core), which leads to anatomical constraints for the final segmentation.

#### 3.1.2. Anisotropic Convolutional Neural Networks

**Figure 2:**
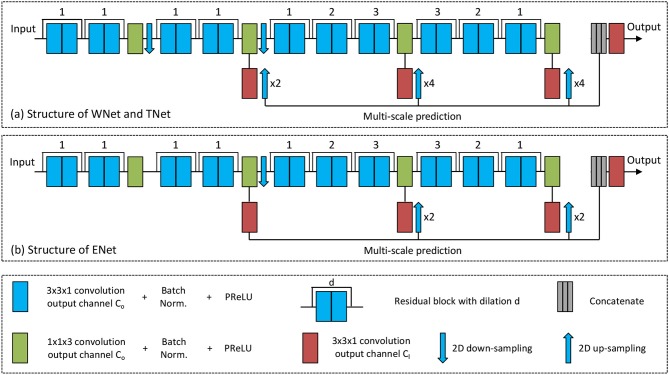
The proposed anisotropic CNNs with residual connection, dilated convolution, and multi-scale prediction. Only one downsampling layer is used in ENet as its input size is smaller.

To achieve a trade-off between memory consumption, model complexity and receptive field for 3D brain tumor segmentation, we propose anisotropic 2.5D CNNs with a large intra-slice receptive field and a relatively small inter-slice receptive field. These CNNs take a stack of slices as input. The receptive field of WNet and TNet is 217 × 217 × 9, and that of ENet is 113 × 113 × 9. Figure 2 shows structures of these proposed CNNs. Note that in previous works (McKinley et al., 2016; Li and Shen, 2017), fusing 2D networks in three orthogonal views was referred to as a 2.5D network, where each of the single-view networks only captures 2D features. In our method, we also use multi-view fusion, but the network in each view is a 2.5D network that captures anisotropic 3D features.

The anisotropic receptive field of our CNNs is achieved by decomposing a typical 3D 3 ×3 × 3 convolution kernel into an intra-slice convolution kernel and an inter-slice convolution kernel, with kernel size of 3 ×3 × 1 and 1 ×1 × 3, respectively. We use four inter-slice convolution layers and 20 intra-slice convolution layers in the backbone of our CNNs, and set the output channel number of these convolution layers to a fixed number *C*_0_. To facilitate the training process, batch normalization is used after each convolution, as shown in green and blue blocks in Figure 2. He et al. (2015) found that Parametric Rectified Linear Units (PReLU) outperforms traditional rectified units, therefore we use PReLU as our activation function. Two 2D downsampling layers are used to reduce the resolution of feature maps of WNet and TNet while avoiding large loss of segmentation details. ENet shares the same structure with WNet and TNet except that it uses only one downsampling layer, as the input size of ENet is smaller.

As shown in Figure 2, intra-slice convolution layers are grouped into 10 blocks, and each block includes two intra-slice convolution layers. To speed the convergence of training, we use residual connections (He et al., 2016) by adding the output of each block directly to its input. We also employ dilated convolution to increase the intra-slice receptive field. The dilation parameter is shown on the top of each residual block in Figure 2. In addition, each CNN uses multi-scale prediction for deep supervision. To get multiple intermediate predictions, three prediction layers with 3 × 3 × 1 convolution are used at different depths of the CNNs, as depicted by red boxes in Figure 2. These intermediate predictions are upsampled to the resolution of the input and concatenated. An additional prediction layer with 3 × 3 × 1 convolution is used to obtain the final score map from the concatenated intermediate predictions. The output channel number of these prediction layers is denoted as *C*_*l*_, and is set to 2 in this paper.

#### 3.1.3. Multi-view Fusion

The above anisotropic CNNs have a small through-plane receptive field, and therefore have a limited ability to make use of 3D contextual information. To overcome this problem, we use multi-view fusion where all WNet, TNet, and ENet are trained in three orthogonal (axial, sagittal, and coronal) views, respectively. At test time, for each network structure, we use the corresponding versions of trained models to obtain the segmentation results in these three views, respectively, and average their softmax outputs to obtain a single fused result.

### 3.2. Augmentation for Training and Testing

Considering the image acquisition process, one underlying anatomy can be observed with different conditions, such as various spatial transformations and intensity noise. Therefore, an acquired image can be seen as only one of many possible observations of the target. Directly applying CNNs to the single observed image may lead the result to be biased toward the specific transformation and noise in the given observation. To address this problem, we predict the segmentation result by considering different spatial transformations and intensity noise for a test image.

Let β denote spatial transformation parameters and *e* represent intensity noise, respectively. Though all images in the BraTS datasets are aligned to a standard orientation, we use rotation, flipping and scaling to augment the variation of local features. Therefore, we represent β as a composition of *r*, *f*_*l*_ and *s*, where *r* denotes the rotation angle along each spatial axis in 3D, *f*_*l*_ is a random binary value representing flipping along each 3D axis or not, and *s* denotes a scaling factor. We consider some prior distributions of these parameters: *r* ~ *U*(0, 2π), *f*_*l*_ ~ *Bern*(0.5), and *s* ~ *U*(0.8, 1.2). In addition, we assume that the intensity noise follows a prior distribution of *e* ~ *N*(0, 0.05) according to Wang et al. (2019a).

To obtain augmented images, we use Monte Carlo simulation to randomly sample β and *e* from the above prior distributions *N* times, and each time we use the sampled parameters to generate a transformed image. The augmentation process is used at both training and testing stage for a given network. For test-time augmentation, the Monte Carlo simulation leads to *N* transformed versions of the same input image, and they are fed into the CNN for inference. We combine the *N* predicted results via majority voting to obtain the final prediction of each structure.

### 3.3. Uncertainty Estimation of Segmentation Results

#### 3.3.1. Voxel-Wise Uncertainty

In our method, the use of test-time augmentation provides multiple prediction results of the same input image with different spatial transformations and intensity changes. The disagreement between these predictions naturally gives an uncertainty estimation of the segmentation. Therefore, we use test-time augmentation to obtain not only segmentation results but also the associated image-based (*aleatoric*) uncertainty. Differently from Wang et al. (2019a), we provide uncertainty estimation not only for the whole tumor, but also for the substructures (tumor core and enhancing tumor core).

To obtain voxel-wise uncertainty estimation, we measure the diversity of the *N* different predictions for a given voxel in the test image. Let *X* and *Y* represent the input image and the output segmentation, respectively, and let *Y*^*i*^ represent the *i*-th voxel's predicted label. Typically, the uncertainty of *Y*^*i*^ can be estimated by the entropy and variance of the distribution of *Y*^*i*^, rather than averaged probability map resulting from *N* Monte Carlo samples that cannot reflect the diversity information. For multi-class segmentation of BraTS, the variance of discrete class label for a voxel is not sufficiently representative. Therefore, we use entropy of *Y*^*i*^ to estimate the voxel-wise uncertainty, which is desired for image segmentation tasks. Assume a set of *N* discrete values (i.e., labels) for *Y*^*i*^ is denoted as $Y^{i}=\left\{y_{1}^{i},y_{2}^{i},\ldots ,y_{N}^{i}\right\}$, then we can approximate the entropy of the distribution of *Y*^*i*^ by:

(1) $$\begin{array}{l} H\left(Y^{i}|X\right)\approx -\overset{M}{\underset{m=1}{\sum }}{\hat{p}}_{m}^{i}\text{ln}\left({\hat{p}}_{m}^{i}\right) \end{array}$$

where ${\hat{p}}_{m}^{i}$ is the frequency of the *m*-th unique value in $Y^{i}$. When $Y^{i}$ is obtained by test-time augmentation with Monte Carlo simulation described in section 3.2, Equation (1) represents voxel-wise *aleatoric* uncertainty.

#### 3.3.2. Structure-Wise Uncertainty

The above Monte Carlo simulation obtains *N* segmentation results for a given structure in a test image. For the *i*-th simulation, let *v*_*i*_ denote the volume of the segmented structure, then the set of volumes of the *N* segmentations is denoted as $V=\left\{v_{1},v_{2},\ldots ,v_{N}\right\}$. Assume that the mean value and standard deviation of V is $\mu_{V}$ and $\sigma_{V}$, respectively. Then the structure-wise uncertainty is estimated as the volume variation coefficient (VVC):

(2) $$\begin{array}{l} VVC=\frac{\sigma_{V}}{\mu_{V}} \end{array}$$

In this paper, V is obtained by test-time augmentation, leading Equation (2) to represent structure-wise *aleatoric* uncertainty.

## 4. Experiments and Results

### 4.1. Data and Implementation Details

We validated our methods with the BraTS 2017^1^ and BraTS 2018^2^ (Menze et al., 2015; Bakas et al., 2017a,b) datasets. The two datasets share the same set of training images from 285 patients, including 75 cases of LGG and 210 cases of HGG. The validation sets of BraTS 2017 and BraTS 2018 contain images from 46 and 66 patients with brain tumors respectively. The testing sets of BraTS 2017 and BraTS 2018 contain images from 146 and 191 patients with brain tumors, respectively. The grades of brain tumors in the validation and testing sets are unknown. Each patient was scanned with FLAIR, T1ce, T1, and T2. The original images were acquired across different views and the resolution was anisotropic. All the images had been re-sampled to an isotropic 1.0 mm × 1.0 mm × 1.0 mm resolution and skull-striped by the organizers. In addition, the four modalities of the same patient had been co-registered. As the BraTS organizers provided ground truth only for the training set, we randomly selected 20% from the training set as our local validation set during training.

Our 2.5D CNNs were implemented in Tensorflow^3^ (Abadi et al., 2016) using NiftyNet^4^,^5^ (Gibson et al., 2018). We used an NVIDIA TITAN X GPU with 12 GB memory, Adaptive Moment Estimation (Adam) (Kingma and Ba, 2014) and Dice loss function (Milletari et al., 2016; Fidon et al., 2017a) for training, with batch size 5, weight decay 10^−7^, initial learning rate 10^−3^, and iteration number 30k. The training patch size was 144 × 144 × 19 for WNet, and 96 × 96 × 19 and 64 × 64 × 19 for TNet and ENet, respectively. We normalized each image by the intensity mean and standard deviation, and set the channel number *C*_*o*_ of intermediate convolution layers to 32 and class number *C*_*l*_ to 2. We trained all WNet, TNet and ENet for axial, sagittal and coronal views separately as our networks had a relatively small number of parameters. Therefore, each network had three different sets of parameters. At test time, the predictions in these three views were averaged. We applied training-time and test-time augmentation to BraTS 2018 dataset according to 3.2, and the Monte Carlo simulation number *N* was set to 20. We uploaded our segmentation results of the validation and testing datasets to the publicly available evaluation server of BraTS 2017 and BraTS 2018, and the server gave quantitative evaluation results in terms of Dice score and Hausdorff distance.

#### 4.1.1. Results of BraTS 2017 Dataset

##### 4.1.1.1. Qualitative results

We first validated our proposed segmentation framework with BraTS 2017 dataset, and test-time augmentation was not used for this experiment. We compared our proposed cascade of anisotropic networks with multi-view fusion with two variants: (1) cascade of 3D isotropic networks that captures 3D features directly, where we remove all 1 × 1 × 3 convolutions in WNet, TNet and ENet, and replace 3 × 3 × 1 convolutions and 2D down-sampling (up-sampling) with 3 × 3 × 3 convolutions and 3D donw-sampling (up-sampling), respectively, and this variant is referred to as isotropic 3D networks; (2) cascade of our anisotropic networks but without multi-view fusion, where the networks are only implemented in axial view, and this variant is referred to as anisotropic 2.5D networks.

**Figure 3:**
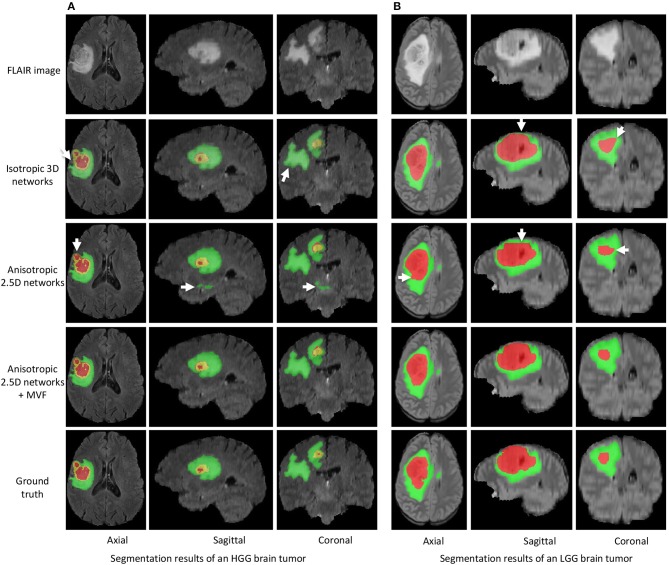
Segmentation results of an HGG brain tumor **(A)** and an LGG brain tumor **(B)** from our local validation set, which is part of BraTS 2017/2018 training set. Edema, non-enhancing tumor core and enhancing tumor core are visualized in green, red, and yellow, respectively. White arrows highlight some mis-segmentations.

Figure 3 shows two examples for HGG and LGG segmentation from our local validation set that is a subset of BraTS 2017/2018 training set. We only show the FLAIR images in the inputs of CNNs for simplicity of visualization. Edema, non-enhancing tumor core and enhancing tumor core are visualized in green, red and yellow, respectively. The results of isotropic 3D networks and anisotropic 2.5D networks are shown in the second and third rows, respectively. In the case of HGG shown in Figure 3A, isotropic 3D networks obtain some mis-segmentations of the edema, and anisotropic 2.5D networks result in some noise in the edema and enhancing tumor core regions. In contrast, the proposed method leads to more accurate segmentation results. Figure 3B shows a case of LGG that does not contain enhancing tumor core. The segmentation results of whole tumor are similar for the three methods. However, the proposed method outperforms isotropic 3D networks and anisotropic 2.5D networks in the tumor core region.

##### 4.1.1.2. Quantitative evaluation

Quantitative evaluation results with the BraTS 2017 validation set are shown in Table 1. The average Dice scores achieved by our method for enhancing tumor core, whole tumor and tumor core are 0.786, 0.905 and 0.838, respectively, which outperforms isotropic 3D networks and anisotropic 2.5D networks. We also compared our method with Kamnitsas et al. (2017a) that uses an ensemble of multiple CNNs for segmentation, and Isensee et al. (2017) that combines 3D U-Net with residual connection and deep supervision. Table 1 shows that our method outperforms the others on the BraTS 2017 validation set. The quantitative evaluation results of our method on BraTS 2017 testing set are shown in Table 2. According to the BraTS 2017 organizers^6^, our method won the second place of the BraTS 2017 segmentation task, while Kamnitsas et al. (2017a) and Isensee et al. (2017) ranked in the first and third place, respectively.

**Table 1:** Dice and Hausdorff distance of our method on validation set of BraTS 2017 (mean ± std).

	**Dice**			**Hausdorff (mm)**		
	**ET**	**WT**	**TC**	**ET**	**WT**	**TC**
Isotropic 3D networks	0.772 ± 0.268	0.885 ± 0.105	0.805 ± 0.196	3.78 ± 5.32	6.73 ± 9.19	7.75 ± 9.98
Anisotropic 2.5D networks	0.741 ± 0.264	0.890 ± 0.076	0.826 ± 0.157	5.32 ± 7.20	12.46 ± 21.47	9.66 ± 14.21
Our method	**0.786** **±** **0.233**	**0.905** **±** **0.066**	**0.838** **±** **0.158**	**3.28** **±** **3.88**	**3.89** **±** **2.79**	**6.48** **±** **8.26**
Kamnitsas et al., 2017a	0.738	0.901	0.797	4.50	4.23	6.56
Isensee et al., 2017	0.732	0.896	0.797	4.55	6.97	9.48

*MVF, multi-view fusion; ET, enhancing tumor core; WT, whole tumor; TC, tumor core. Our method: cascaded framework with anisotropic 2.5D CNNs and MVF. Bold value shows the best performance*.

**Table 2:** Dice and Hausdorff distance of our method on testing set of BraTS 2017 (mean ± std).

	**Dice**			**Hausdorff (mm)**		
	**ET**	**WT**	**TC**	**ET**	**WT**	**TC**
Our method	**0.783** **±** **0.222**	0.874 ± 0.132	0.775 ± 0.270	**15.90** **±** **67.86**	6.55 ± 10.69	27.05 ± 84.43
Kamnitsas et al., 2017a	0.729	**0.886**	**0.785**	36.0	**5.01**	**23.10**
Isensee et al., 2017	0.647 ± 0.326	0.858 ± 0.161	0.775 ± 0.269	–	–	–

*ET, enhancing tumor core; WT, whole tumor; TC, tumor core. Bold value shows the best performance*.

#### 4.1.2. Results of BraTS 2018 Dataset

We then applied our proposed segmentation framework to BraTS 2018 dataset. To validate the effect of test-time augmentation (TTA), we compared three network configurations as underpinning CNNs: (1) 3D UNet (Abdulkadir et al., 2016) reimplemented by NiftyNet, (2) our cascaded networks where the whole tumor, tumor core and enhancing tumor core were segmented by WNet, TNet, and ENet, respectively, and (3) adapting WNet for multi-class segmentation without using a cascade of binary predictions, where we changed the output channel number for prediction layers to 4. We refer to this variant as multi-class WNet and also use multi-view fusion for it. The 3D U-Net and multi-class WNet were trained in the same way as our cascaded networks.

##### 4.1.2.1. Qualitative results

**Figure 4:**
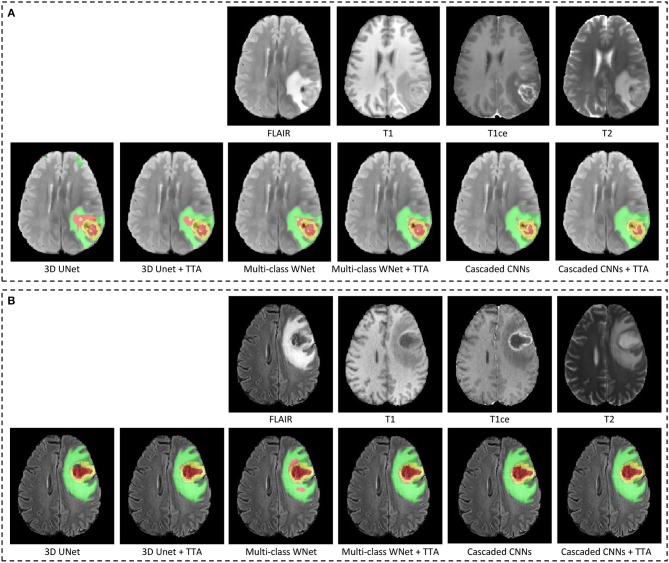
Examples of test-time augmentation (TTA) combined with different CNNs for brain tumor segmentation. The images are from BraTS 2018 validation set, of which ground truth are not provided by the organizer. In each subfigure, the first row shows the input image of the same patient in four modalities, and the second row shows segmentation results. Edema, non-enhancing tumor core and enhancing tumor core are visualized in green, red, and yellow, respectively. **(A,B)** Show images of two different patients.

Figure 4 shows two examples from the BraTS 2018 validation set. In each subfigure, the input images (FLAIR, T1, T1ce, and T2) are shown in the first row and the segmentation results of different networks with and without TTA are presented in the second row. In Figure 4A, the result of 3D UNet without TTA contains some false positives in the edema and non-enhancing tumor core regions. In contrast, the result of 3D UNet + TTA is more spatially consistent. The result obtained by multi-class WNet without TTA also contains some noise for the segmented non-enhancing tumor core, and multi-class WNet + TTA obtains a smoother segmentation. It can also be observed that our cascaded CNNs + TTA performs better on the tumor core than the counterpart without TTA. In Figure 4B, 3D UNet seems to obtain an under-segmentation in the central part of the tumor core, and 3D UNet + TTA overcomes this under-segmentation. Multi-class WNet without TTA seems to have an over segmentation for the non-enhancing tumor core region, and the counterpart with TTA achieves a higher accuracy in contrast. For our cascaded CNNs, TTA also helps to improve the spatial consistency of the segmentation result in this case.

##### 4.1.2.2. Quantitative evaluation

Table 3 shows the quantitative evaluation results of different approaches on the validation set of BraTS 2018. Dice scores achieved by 3D UNet without TTA for enhancing tumor core, whole tumor and tumor core are 0.734, 0.864 and 0.766, respectively. Combining TTA with 3D UNet achieved a better performance, leading to Dice scores of 0.754, 0.873, and 0.783 for these structures, respectively. Applying test-time augmentation to multi-class WNet and the cascaded networks also leads to an improvement of segmentation accuracy. We also compared our method with Myronenko (2018) and Isensee et al. (2018) that ranked the first and second of BraTS 2018 segmentation challenge, respectively^7^. Myronenko (2018) used an ensemble of 10 models, and we list the result of a single model and that of model ensemble reported by Myronenko (2018). Isensee et al. (2018) trained a 3D U-Net with additional datasets for the segmentation task. It can be observed that our method performs closely to these two compared methods on BraTS 2018 validation set. Quantitative evaluation results of our cascaded CNNs with TTA on BraTS 2018 testing set is presented in Table 4. The results are compared with those of Myronenko (2018) and Isensee et al. (2018). Note that Myronenko (2018) requires a large amount of GPU memory (32 GB) for training, and Isensee et al. (2018) trained the model with additional datasets. Table 4 shows that the segmentation accuracy of our proposed framework is comparable with that of the other two counterparts.

**Table 3:** Dice and Hausdorff distance of different methods on validation set of BraTS 2018 (mean ± std).

	**Dice**			**Hausdorff (mm)**		
	**ET**	**WT**	**TC**	**ET**	**WT**	**TC**
3D UNet	0.734 ± 0.284	0.864 ± 0.146	0.766 ± 0.230	9.37 ± 22.95	12.00 ± 21.22	10.37 ± 13.47
3D UNet + TTA	0.754 ± 0.263	0.873 ± 0.125	0.783 ± 0.168	4.53 ± 9.60	5.90 ± 6.80	8.03 ± 10.31
Multi-class WNet	0.757 ± 0.257	0.890 ± 0.089	0.725 ± 0.245	4.24 ± 7.97	4.99 ± 6.53	12.13 ± 13.41
Multi-class WNet + TTA	0.771 ± 0.242	0.896 ± 0.071	0.730 ± 0.255	4.44 ± 8.20	4.92 ± 6.42	11.13 ± 13.46
Cascaded networks	0.792 ± 0.233	0.903 ± 0.057	0.854 ± 0.142	3.34 ± 4.15	5.38 ± 9.31	6.61 ± 8.55
Cascaded networks + TTA	0.797 ± 0.229	0.902 ± 0.056	0.858 ± 0.139	3.13 ± 3.78	6.18 ± 9.53	6.37 ± 8.19
Cascaded networks + TTA + CRF0	0.803 ± 0.228	0.905 ± 0.056	0.862 ± 0.136	3.09 ± 3.75	5.97 ± 8.22	6.25 ± 7.87
Cascaded networks + TTA + CRF1	0.807 ± 0.225	0.908 ± 0.054	**0.869** **±** **0.126**	3.01 ± 3.69	5.86 ± 8.16	**6.09** **±** **7.74**
Myronenko, 2018 (single model)	0.815	0.904	0.860	3.80	**4.48**	8.28
Myronenko, 2018 (ensemble)	**0.823**	**0.910**	0.867	3.93	4.52	6.85
Isensee et al., 2018	0.810	0.908	0.854	**2.54**	4.97	7.04

*ET, enhancing tumor core; WT, whole tumor; TC, tumor core; TTA, test-time augmentation. CRF0: naive conditional random field for post-processing. CRF1: our uncertainty-aware conditional random field. Bold value shows the best performance*.

**Table 4:** Dice and Hausdorff evaluation of our cascaded CNNs with test-time augmentation (TTA) on testing set of BraTS 2018 (mean ± std).

	**Dice**			**Hausdorff (mm)**		
	**ET**	**WT**	**TC**	**ET**	**WT**	**TC**
Cascaded networks + TTA	0.747 ± 0.259	0.878 ± 0.119	0.796 ± 0.250	4.16 ± 7.07	5.97 ± 8.56	6.71 ± 10.27
Myronenko, 2018	0.766 ± 0.256	0.884 ± 0.118	0.815 ± 0.250	3.77 ± 8.61	5.90 ± 10.01	4.81 ± 7.52
Isensee et al., 2018	0.779 ± 0.239	0.878 ± 0.129	0.806 ± 0.250	2.90 ± 3.85	6.03 ± 9.98	5.08 ± 8.09

*ET, enhancing tumor core; WT, whole tumor; TC, tumor core. Myronenko (2018) used an ensemble of 10 models for the segmentation*.

##### 4.1.2.3. Uncertainty estimation

**Figure 5:**
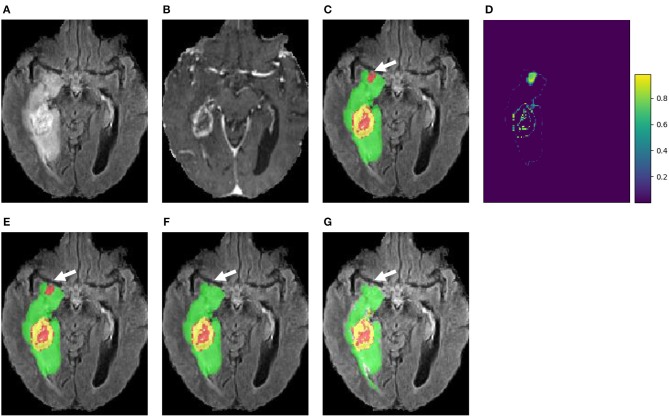
An example of brain tumor segmentation result and the associated voxel-wise uncertainty estimation based on our cascaded CNNs with test-time augmentation (TTA). Taking the uncertainty information for post-processing by conditional random field (CRF) helps to correct the mis-segmented region, as shown in **(F)**. **(A)** FLAIR, **(B)** T1ce, **(C)** Initial segmentation, **(D)** Voxel-wise uncertainty, **(E)** Post-process with CRF, **(F)** Post-process with uncertainty-aware CRF, and **(G)** Ground truth.

Figure 5 presents a case from our local validation set of BraTS 2018, where Figures 5C,D show the results of our cascaded CNNs and the corresponding voxel-wise uncertainty obtained by TTA, respectively. It can be observed that most uncertain results concentrate on the border of the tumor's substructures and some regions that are potentially mis-segmented. The white arrow in Figure 5C highlights a region that has been mis-segmented by CNNs, and the corresponding region has high uncertainty values in Figure 5D. To investigate the usefulness of the uncertainty information for improving segmentation accuracy, we reset the foreground and background probability of voxels with uncertainty higher than a threshold value (i.e., 0.2) to 0.5, and then use a conditional random field (CRF) for post-processing. This method is referred to as uncertainty-aware CRF, and it is compared with a naive CRF that is applied to the probability output of CNNs directly. Figures 5E,F show that the uncertainty-aware CRF outperforms the naive CRF for post-processing. Table 3 shows a quantitative comparison between these post-processing methods using and not using uncertainty information on validation set of BraTS 2018.

**Figure 6:**
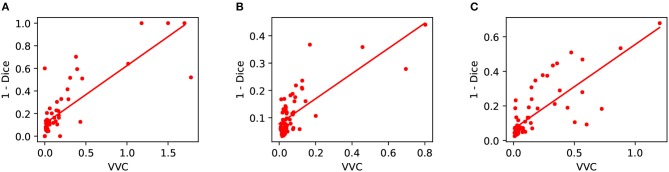
Relationship between segmentation error (1-Dice) and structure-wise uncertainty in terms of volume variation coefficient (VVC) for BraTS 2018 validation set. **(A)** Enhancing core, **(B)** Whole tumor, and **(C)** Tumor core.

We also measured structure-wise uncertainty based on VVC defined in Equation (2) for BraTS 2018 validation set. Figure 6 shows the relationship between structure-wise segmentation error in terms of 1-Dice and uncertainty in terms of VVC. The figure shows that for all the three structures of enhancing tumor core, whole tumor and tumor core, a higher VVC value tends to be linked with a higher segmentation error. This demonstrates that the structure-wise uncertainty based on our test-time augmentation is informative and it can indicate potential mis-segmentations.

## 5. Discussion and Conclusion

The proposed cascaded system is well-suited for hierarchical tumor region segmentation. Compared with using a single network for multi-class segmentation, its main advantages are: (1) The use of three binary segmentation networks decomposes the complex task of multi-class segmentation and allows for a simpler network for each sub-task. They reduce the risk of over-fitting and are easier to train. (2) The cascade can effectively reduce the number of false positives because a subsequent network (e.g., TNet) only works on the image region selected by its precedent network (e.g., WNet). (3) The decomposition of the segmentation task also imposes strong spatial constraints which follows the anatomical structures of the brain tumor. It is also possible to model the hierarchical nature of the labels by adopting task-specific loss functions (e.g., Fidon et al., 2017a). However, Fidon et al. (2017a) did not use the hierarchical structural information as spatial constraints. Unlike most works that optimize the segmentation based on mutually exclusive edema, necrotic, and enhancing tumor core, our method optimizes the hierarchical whole tumor, tumor core and enhancing tumor core. This leads to the idea of training networks on such loss criteria to simultaneously obtain these hierarchical structures in a single forward pass, as demonstrated by Myronenko (2018). For some clinical cases where the tumor does not have edema component, i.e., the region of whole tumor is the same as that of tumor core, our model may encounter some difficulties (e.g., false positives of edema) as all the training data in our experiments include edema region. However, as our WNet segments the edema region and tumor core region as a whole, the tumor core region in such cases will not be missed in the output of WNet. It is of interest to validate the proposed method on such cases in the future. In addition, in our cascaded segmentation framework, segmentation of whole tumor (tumor core) was used as a crisp mask for tumor core (enhancing tumor core), this may lead mis-segmentations in an early stage to cause mis-segmentations in a later stage. It would be of interest to investigate a better solution to combine the results obtained in different stages.

Compared with the single multi-class network approach using similar network structures, the training and inference of our proposed cascade require a longer time. In practice, we found that it is not a critical issue for automatic brain tumor segmentation. In fact, the inference of our method is more efficient than many competitive approaches such as DeepMedic (Kamnitsas et al., 2017b) and ScaleNet (Fidon et al., 2017b).

The multi-view fusion is an important component of the proposed system (as demonstrated in Figure 3). It is designed to combine the outputs from the lightweight and anisotropic networks applied in different views so that the 3D contextual information is fully utilized. To further incorporate different imaging resolutions in the multi-view fusion, it might be helpful to consider a weighted combination of the orthogonal views rather than a simple arithmetic mean (Mortazi et al., 2017).

From Table 3 we find that the improvement obtained by TTA varies for different networks. For 3D UNet (Abdulkadir et al., 2016), the performance improvement is considerable, especially for the Hausdorff distance. For our cascaded networks, the improvement is relatively smaller but TTA is also effective to reduce the distance errors for enhancing tumor and tumor core. Table 3 also shows that TTA reduces the standard deviation (improves the robustness) of the networks in most cases, especially for 3D UNet. For our cascaded networks, the standard deviations for enhancing tumor and tumor core are also smaller when TTA is used. Therefore, TTA can be seen as a robustness booster. In the proposed system, data augmentation only includes adding random intensity noise and spatial transformations such as rotation, flipping and scaling. It is also possible to adopt more complex transformations such as elastic deformations (Abdulkadir et al., 2016).

We have investigated the test image-based (*aleatoric*) uncertainty for brain tumor segmentation using test-time augmentation. We additionally show that the uncertainty information can be leveraged to improve the segmentation accuracy, as demonstrated in Table 3 and Figure 5. The obtained uncertainty could be useful for downstream analysis such as uncertainty-aware volume measurement (Eaton-Rosen et al., 2018) and guiding user interactions (Wang et al., 2018b). Combining *epistemic* uncertainty based on test-time dropout or CNN ensembles (Kamnitsas et al., 2017a; Myronenko, 2018) and *aleatoric* uncertainty based on test-time augmentation is also an interesting future direction. It should be noticed that current methods for BraTS challenge heavily rely on voxel-wise annotations, which is difficult and time-consuming to collect for large datasets. In the future, it is of interest to learn from weakly or partially annotated brain tumor images in a larger dataset and improve generalizability of the CNNs. Some of the automatically segmented results can also be interactively refined to improve the robustness of brain tumor segmentation for clinic use (Wang et al., 2019b).

In conclusion, we have developed a novel system consisting of a cascade of 2.5D CNNs for brain tumor segmentation from multi-modal MRI, which decomposes the multi-class segmentation task into three sequential binary segmentation tasks. The 2.5D CNNs consider the balance between memory consumption, model complexity and recpetive field, and are combined with multi-view fusion for robust segmentation. We also studied the effect of combining test-time augmentatiofn with CNNs in the segmentation task and investigated the resulting *aleatoric* uncertainty estimation for the segmentation results. Experimental results based on BraTS 2017 dataset showed that our method was one of the top-performing methods. Experiments also showed that test-time augmentation led to an improvement of segmentation accuracy for different CNN structures and effectively obtained voxel-wise and structure-wise uncertainty estimation of the segmentation results that helps to improve segmentation accuracy.

## Data Availability

Publicly available datasets were analyzed in this study. This data can be found here: https://www.med.upenn.edu/sbia/brats2018/data.html.

## Author Contributions

GW, WL, and TV contributed conception and design of the study. GW and WL contributed implementation of the method. GW conducted the experiments and wrote the manuscript. All authors contributed to manuscript revision, proofreading, and approved the submitted version.

### Conflict of Interest Statement

WL was employed by King's College London during most of the preparation of this work and was employed by company NVDIA for the final editing and proofreading of the manuscript. SO is a founder and shareholder of BrainMiner Ltd, UK. The remaining authors declare that the research was conducted in the absence of any commercial or financial relationships that could be construed as a potential conflict of interest.

## Footnotes

^1^http://www.med.upenn.edu/sbia/brats2017.html

^2^http://www.med.upenn.edu/sbia/brats2018.html

^3^https://www.tensorflow.org

^4^http://niftynet.io

^5^https://github.com/NifTK/NiftyNet/tree/dev/demos/BRATS17

^6^https://www.med.upenn.edu/sbia/brats2017/rankings.html

^7^https://www.med.upenn.edu/sbia/brats2018/rankings.html
